# Clinical pharmacist intervention to improve medication safety for hip fracture patients through secondary and primary care settings: a nonrandomised controlled trial

**DOI:** 10.1186/s13018-023-03906-2

**Published:** 2023-06-13

**Authors:** Ben Tore Henriksen, Maria Krogseth, Randi Dovland Andersen, Maren Nordsveen Davies, Caroline Thy Nguyen, Liv Mathiesen, Yvonne Andersson

**Affiliations:** 1 Research Department, Hospital Pharmacies Enterprise, South Eastern Norway, Tonsberg, Norway; 2 Division of Surgery, Vestfold Hospital Trust, Tonsberg, Norway; 3 Department of Pharmacy, Faculty of Mathematics and Natural Sciences, University of Oslo, Oslo, Norway; 4 Old Age Psychiatry Research Network, Telemark Vestfold, Vestfold Hospital Trust, Tonsberg, Norway; 5 Department of Research, Telemark Hospital Trust, Skien, Norway; 6 Institute of Health and Society, Research Centre for Habilitation and Rehabilitation Models and Services (CHARM), Faculty of Medicine, University of Oslo, Oslo, Norway; 7 Department of Pharmacy, Faculty of Health Sciences, UiT The Arctic University of Tromso, Tromso, Norway

**Keywords:** Medication reconciliation, Medication safety, Pharmacists, Patient safety, Transitions in care

## Abstract

**Background:**

Hip fracture patients face a patient safety threat due to medication discrepancies and adverse drug reactions when they have a combination of high age, polypharmacy and several care transitions. Consequently, optimised pharmacotherapy through medication reviews and seamless communication of medication information between care settings is necessary. The primary aim of this study was to investigate the impact on medication management and pharmacotherapy. The secondary aim was to evaluate implementation of the novel Patient Pathway Pharmacist intervention for hip fracture patients.

**Methods:**

Hip fracture patients were included in this nonrandomised controlled trial, comparing a prospective intervention group (*n* = 58) with pre-intervention controls who received standard care (*n* = 50). The Patient Pathway Pharmacist intervention consisted of the steps: (A) medication reconciliation at admission to hospital, (B) medication review during hospitalisation, (C) recommendation for the medication information in the hospital discharge summary, (D) medication reconciliation at admission to rehabilitation, and (E) medication reconciliation and (F) review after hospital discharge. The primary outcome measure was quality score of the medication information in the discharge summary (range 0–14). Secondary outcomes were potentially inappropriate medications (PIMs) at discharge, proportion receiving pharmacotherapy according to guidelines (e.g. prophylactic laxatives and osteoporosis pharmacotherapy), and all-cause readmission and mortality.

**Results:**

The quality score of the discharge summaries was significantly higher for the intervention patients (12.3 vs. 7.2, *p* < 0.001). The intervention group had significantly less PIMs at discharge (− 0.44 (95% confidence interval − 0.72, − 0.15), *p* = 0.003), and a higher proportion received prophylactic laxative (72 vs. 35%, *p* < 0.001) and osteoporosis pharmacotherapy (96 vs. 16%, *p* < 0.001). There were no differences in readmission or mortality 30 and 90 days post-discharge. The intervention steps were delivered to all patients (step A, B, E, F = 100% of patients), except step (C) medication information at discharge (86% of patients) and step (D) medication reconciliation at admission to rehabilitation (98% of patients).

**Conclusion:**

The intervention steps were successfully implemented for hip fracture patients and contributed to patient safety through a higher quality medication information in the discharge summary, fewer PIMs and optimised pharmacotherapy.

*Trial registration*: NCT03695081.

**Supplementary Information:**

The online version contains supplementary material available at 10.1186/s13018-023-03906-2.

## Key points

- This study demonstrated an innovative and feasible intervention, a Patient Pathway Pharmacist, for hip fracture patients to ensure medication safety in both secondary and primary care settings.
- The study showed improved communication of medication information and optimised pharmacotherapy through the whole patient journey.
- This model may serve as a contribution to future practice and healthcare policy design.

**Supplementary Information:**

The online version contains supplementary material available at 10.1186/s13018-023-03906-2.

## Introduction

Hip fracture is a serious incident for the individual as it may lead to disability, increased care needs, reduced quality of life, and is associated with high mortality [1, 2]. Hip fractures also have large impact on the healthcare services and society. To illustrate, hip fractures affected more than 14 million patients in 2019 [3], and the number will likely increase in the future with the ageing population [4–6]. In the UK, there were 76,000 hip fracture patients [7], with a yearly hospital cost estimated to £1.1 billion [8, 9]. A typical hip fracture patient is an older adult with multimorbidity and polypharmacy (i.e. using five or more medications), who experience several care transitions after the fracture incident [5, 10–13].

Care transitions between home, hospital, and rehabilitation institution are crucial in healthcare, yet they pose a significant patient safety risk [14–16]. For many patients, information is conveyed only through written summaries from the previous care setting. Discharge summaries, for example, may be the only source for medication information after hospitalisation [15, 17, 18]. Therefore, the discharge summary needs to contain complete information about all medications, including any changes, to ensure appropriate treatment in primary care [15, 18]. Nevertheless, discharge summaries are often of poor quality, with hip fracture patients being no exception [19, 20]. Thus, important medication information may be missing for the clinician taking over responsibility for patients’ care [21]. Polypharmacy increase the risk of low quality discharge summaries and higher number of potentially inappropriate medications (PIMs) [20, 22]. One type of PIM is medications that increase the risk of falling, and have been detected in 90% of hip fracture patients [23]. Despite polypharmacy, hip fracture patients may lack necessary medications, e.g. osteoporosis therapy after the low-energy fracture (i.e. anti-bone resorptive agents, such as bisphosphonates) [9]. The totality of these risk factors may harm patients by potentially causing medication errors [24], new falls and fractures [23], and increased mortality [21, 25, 26].

For hip fracture patients, the combination of the above-mentioned evidence reflects the need for seamless communication of a clear and correct medication list, containing appropriate pharmacotherapy and the plan for follow-up. We developed an innovative, multi-step, clinical pharmacist intervention specifically for the hip fracture patient. The pharmacist followed the patient throughout the healthcare system; both within and between care settings—i.e. a Patient Pathway Pharmacist. To the best of our knowledge, a clinical pharmacist service where the pharmacist is allocated to a specific patient pathway has not been described previously. The intention was to secure safe and optimised pharmacotherapy in every care setting by performing repeated medication reconciliations, reviews and assisting in communication between care settings. The intervention would also be in line with recommendations for continuity of healthcare providers in coordination of medication management with multidisciplinary teams [27, 28]. Previous studies have included clinical pharmacists in discharge management and care transitions, which resulted in optimised medication information and reduced discrepancies [11, 29–32]. Through the medication reviews, clinical pharmacists have contributed to optimised pharmacotherapy by reducing the number of PIMs, suggested to start recommended therapies, and increasing medication appropriateness [33–35]. Furthermore, clinical pharmacist interventions have also shown to reduce readmissions and mortality [36, 37].

Thus, the primary aim of the study was to investigate the impact of the Patient Pathway Pharmacist intervention for hip fracture patients on medication management and pharmacotherapy compared to pre-intervention controls. The secondary aim was to evaluate the implementation of the Patient Pathway Pharmacist intervention for hip fracture patients in primary and secondary care settings.

## Methods

### Study design

This study compared a prospective intervention group with pre-intervention controls, and was conducted in a region in South-Eastern Norway (approximate population of 250,000). The study (ClinicalTrials.gov ID: NCT03695081) is reported using the Consolidated Standards of Reporting Trials [38].

### Sample and setting

Hip fracture patients ≥ 18 years of age who were admitted to a Norwegian public regional hospital and followed an in-hospital standardised fast track procedure for hip fracture management could be included in the study. Patients were included irrespective of residence, with both high- and low-energy fractures. The in-hospital fast track was a procedure describing patient flow and healthcare professionals’ responsibility through all hospital settings. Exclusion criteria were terminally ill patients (life expectancy less than one week) and non-fast track patients (e.g. pathological fracture and fracture in already hospitalised patients). After discharge, a majority of patients received initial rehabilitation care in an institution prior to returning to their habitual accommodation with or without district (home care) nursing services, or a prolonged nursing home stay. The great majority of the Norwegian healthcare system is public with universal access.

The intervention group were patients admitted from 03 September 2018 to 04 April 2019. The patient last admitted was assessed for eligibility without any details except name, date of birth, and time of admission to avoid selection bias. The control group constituted a retrospective sample of hip fracture patients randomly selected using a random number generator (Mersenne Twister, Microsoft Excel 2016). Patients discharged from the hospital within the three pre-intervention months (01 June–31 August 2018) were eligible as controls. As control patients were included retrospectively and the primary outcome was discharge summary score (described below), patients who died during hospitalisation were excluded from this group.

#### The Patient Pathway Pharmacist intervention group

**Fig. 1:**
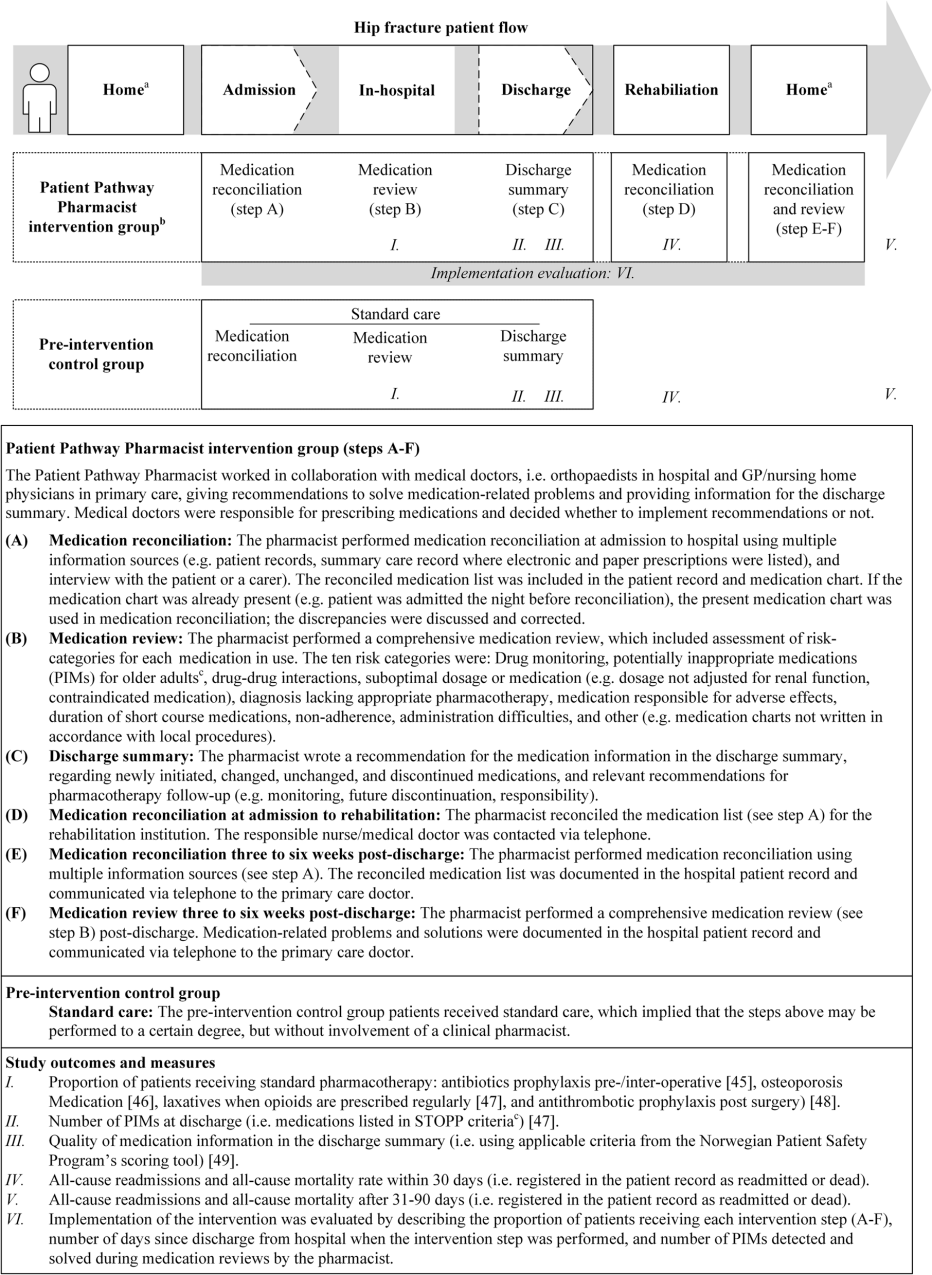
Graphical depiction of the Patient Pathway Pharmacist study. The Patient Pathway Pharmacist steps per protocol (**A**–**F**), standard care steps, and study outcomes and measurement (**I**–**VI**) (figure inspired by Lea et al.[36] and Perera et al.[44], adapted from Henriksen et al.[10]). ^a^Private home, care home, or nursing home. ^b^Following the Integrated Medicines Management method. ^c^PIMs were defined by using the STOPP criteria, version 2

We developed a clinical pharmacist intervention, the Patient Pathway Pharmacist, based on the Integrated Medicines Management method adapted for a Norwegian setting [39–43] and feedback from stakeholders [10]. The pharmacist intervened at six longitudinal points in secondary and primary care settings (Fig. 1). The pharmacist performed medication reconciliation at admission to the hospital (step A), a medication review during the hospital stay (step B), and wrote a medication information for inclusion in the discharge summary (step C). The pharmacist performed new medication reconciliations at admission to rehabilitation (step D) and 3–6 weeks post-discharge (step E), and performed a new medication review 3–6 weeks post-discharge (step F). For all steps, the Patient Pathway Pharmacist collaborated with the responsible medical doctor (often an orthopaedist in hospital, and GP or nursing home physician in primary care). The pharmacist advised how to solve medication-related discrepancies or problems, and the responsible medical doctor decided whether to implement recommendations or not. Each step is described in more detail in Fig. 1.

#### Control group

The control group patients received standard care in accordance with local procedures. This implied that the admitting medical doctor in the emergency care unit was responsible for medication reconciliation. The medical doctor responsible for discharge, most often a medical doctor at the orthopaedic ward, wrote the discharge summary, including medication information. Medication reviews were not mandatory, but some patients may have received medication review as a part of hospital care, e.g. geriatric supervision (Fig. 1). The medication management tasks were performed without assistance from a clinical pharmacist.

### Study outcomes

The primary and secondary outcomes are related to the primary aim, whilst the implementation evaluation is related to the secondary aim.

#### Primary aim

The primary outcome was a quality score of the medication information in the discharge summary, using applicable criteria from the Norwegian Patient Safety Program’s scoring tool [49]. The following seven criteria were assessed; (generic) names, formulation, dose, frequency, indication, reason for changes, and category for changed medication (i.e. new, stopped, changed or short course). The score for each criteria was based on whether the information was present for all medications (two points), for at least one medication (one point), or not at all (zero points), hence the final score ranged from 0 to 14. The discharge summaries were independently rated by two experienced clinical pharmacists (intervention group) or an MSc Pharmacy master student and an experienced clinical pharmacist (control group). We used the average score between raters as the primary outcome measure. The inter-rater reliability score was excellent for the intervention group (Intraclass Correlation Coefficient, two-way mixed effect model, absolute agreement, average measure (ICC) 0.96 (95% confidence interval (CI) 0.93, 0.98), *p* < 0.001) and good for the control group (ICC 0.83 (95% CI 0.71, 0.91), *p* < 0.001) [50]. For detailed reliability test results and specific criteria assessed in the discharge summary scoring tool, see Additional file 1.

The secondary outcomes were PIMs at discharge adjusted for PIMs at admission using Analysis of covariance (ANCOVA); standard pharmacotherapy; and all-cause readmission and mortality, 30 and 90 days post-discharge. PIMs were defined as medications listed in the Norwegian translation of the screening tool of older people’s prescriptions criteria, version 2, for patients 65 years or older (STOPP) [47, 51]. Only STOPP criteria that could be assessed by clinical data available in patient records were included, causing 24 of the total 81 categories to be excluded (Additional file 2: Table S3). Post hoc, we added a secondary exploratory outcome for proportion of patients receiving standard pharmacotherapy in accordance with local procedures during hip fracture hospitalisation. These procedures reflected standards in international guidelines in the treatment of hip fracture patients and included antibiotics prophylaxis pre-/interoperative [45], osteoporosis medication [46], laxatives when opioids are prescribed regularly [47], and antithrombotic prophylaxis post-surgery [48]. The medications needed to be documented in the patient record or medication chart.

#### Secondary aim

To evaluate implementation of the Patient Pathway Pharmacist intervention we used the following process measures: proportion of patients receiving the intervention according to protocol in each of the steps A–F (Fig. 1), number of days since discharge from hospital when the intervention step was performed, and number of PIMs detected and solved during medication reviews by the Patient Pathway Pharmacist.

### Data collection

We collected data from hip fracture patients’ hospital records and medication charts on patient demographics (e.g. age, sex, living situation), clinical measures (e.g. acute and chronic diagnoses, Charlson’s Comorbidity Index [52], complications during hospitalisation, readmissions, and deaths), process measures (e.g. days in hospital), medication management characteristics (e.g. documented medication reconciliation and review) and outcome measures (e.g. discharge summary information, PIMs, standard pharmacotherapy, readmissions, and mortality). Blinding was impossible due to the nature of the intervention.

### Sample size calculation

We used information from a study using a previous version of the score tool [19] and, with expert opinion and informal testing, estimated a mean discharge summary score difference of six (SD three) with significance level (*α*) of 5% and power (*β*) of 80%, resulting in an estimated total number of eight participants. We wanted to include as many patients as possible to contribute to the secondary outcomes. Thus, the total number of patients included in both groups were decided to be 110, limited by resources.

### Statistical analysis

Data were managed with EpiData, v4.6.0.2 [53], and analysed with Stata software, v16.1 [54]. Data were presented using mean, SD and 95% CI if normally distributed data, and median and interquartile range (IQR: 25th and 75th percentile) if skewed. For hypothesis testing, t-tests were used for normally distributed data and nonparametric tests for differences in ranks for non-normally distributed data that failed transformation. For nominal variables, we tested for differences in frequency using Fisher’s exact test or Pearson’s Chi Square-test. ANCOVA was used to compare changes in PIMs between the intervention and the control group, with PIMs at admission as a covariate. Two-sided *p*-values ≤ 0.01 were considered statistically significant. Statistical analyses were performed in collaboration with a statistician and all tests met appropriate assumptions.

### Ethics approval and consent to participate

The Regional Committee for Medical and Health Research Ethics in South East Norway found the study to be outside the Norwegian Health Research Act (ref ID: 2017/2172, 20 December 2017). In accordance with Norwegian law, the study was approved by Norwegian Centre for Research Data (ref ID: 556662 and 359479), the Data Protection Official for Vestfold Hospital Trust at the time the study was conducted. All patients, or their next-of-kin, in the intervention group gave their informed and written consent, in accordance with approval. The control patients were exempted consent in accordance with approval. This study was performed in line with the principles of the Declaration of Helsinki.

## Results

**Fig. 2:**
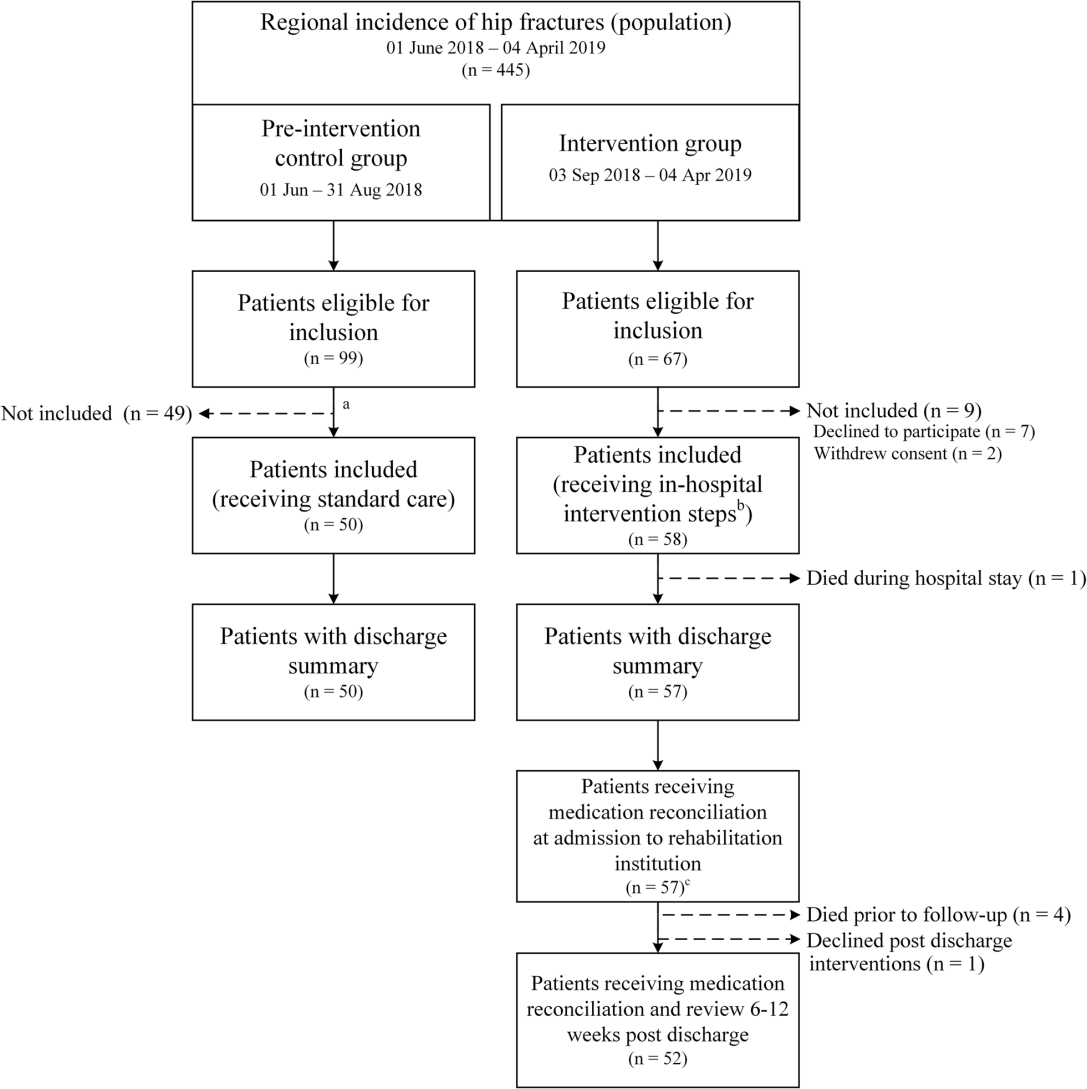
Flow chart illustrating the control group (standard care), and the Patient Pathway Pharmacist intervention group. ^a^Patients were randomly selected for inclusion. ^b^The in-hospital intervention steps were medication reconciliation at admission, medication review during hospital stay, and a patient record document containing the medication section for the discharge summary. ^c^One patient did not receive medication reconciliation for an unknown reason

A total of 108 patients were included; 58 patients in the intervention group and 50 in the pre-intervention control group (Fig. 2). The mean age was 84 years in both groups, and nearly all patients were 65 years or older (*n* = 56/58 in intervention group and *n* = 48/50 in control group). There were no significant differences in characteristics or number of medications at admission (Table 1). A majority of patients (*n* = 84/108) used five medications or more at hospital admission (47/58 in the intervention group vs. 37/50 control group). The number of medications during hospitalisation increased with a mean of 3.8 (95% CI 3.1, 4.5, *p* < 0.001) and 2.5 (95% CI 1.8, 3.2, *p* < 0.001) for the intervention and control group respectively.

**Table 1:** Characteristics of included patients

Variable	Intervention, *n* = 58	Control, *n* = 50	*P*-value^a^
Female, *n* (%)	36 (62)	26 (52)	0.291^b^
*Age, years*			
Mean (SD)	84 (10)	84 (9)	0.981^c^
Range	50–104	56–99	
*Living situation (prior to hospitalisation),* *n* (%)			
Home with/without district nursing service	47 (81)	32 (64)	0.129^b^
Care home with district nursing service	5 (9)	7 (14)	
Nursing home	6 (10)	11 (22)	
*Responsible for medication administration*, *n* (%)			
Patient	32 (55)	19 (38)	0.190^d^
Other than patient	23 (40)	27 (54)	
No medication	3 (5)	4 (8)	
*Comorbidity measured using CCI*^f^			
Median (IQR)	1 (0–2)	2 (1–3)	0.094^e^
Range	0–9	0–7	
Type of hip fracture, *n* (%)			
Femoral Neck Fracture	29 (50)	26 (52)	0.942^d^
Trochanteric Fracture	27 (47)	22 (44)	
Subtrochanteric Fracture	2 (3)	2 (4)	
*Days in hospital*			
Median (IQR)	4 (3–6)	5 (4–8)	0.016^e^
Range	1–22	2–19	
*Complications*^g^, *n*			
Median (IQR)	0 (0–1)	0.5 (0–1)	0.706^e^
Range	0–6	0–3	
*Total number of medications*			
At admission, mean (SD)	8.1 (4.9)	7.7 (4.4)	0.672^c^
At discharge, mean (SD)	11.8 (3.8)	10.2 (3.6)	0.026^c^

*CCI*—Charlson’s Comorbidity Index, *IQR*—interquartile range (25th and 75th percentile), *SD*—standard deviation

^a^*P*-value for differences between control group and intervention group given by ^b^Pearson’s Chi Square-test

^c^Independent two-sample student’s t-test

^d^Fisher’s exact test

^e^Mann–Whitney *U*-test

^f^Each patient was given a score in accordance with the original Charlson’s Comorbidy Index to indicate comorbidity burden and predict mortality risk (e.g. one year mortality risk for zero points (i.e. no comorbidity) = 12%, and ≥ 5 points = 85%); examples of one point for myocardial infarct or dementia, two points for any tumour, three points for moderate/sever liver disease, six points for metastatic solid tumour [52]

^g^Complications during hospitalisation were: delirium, decubitus, deterioration of heart or lung disease, falls during hospital stay, hypoxia, infection, thromboembolism, transfusion of blood, and post-operative invasive analgesia

### Impact of the Patient Pathway Pharmacist intervention on outcome measures

The mean discharge summary score was 12.3 (95% CI 11.7, 12.9) in the intervention group, compared to 7.2 (95% CI 6.7, 7.7) in the control group (*p* < 0.0001) (Table 2). The sub-scores for each criteria also were significantly higher in the intervention group, with median score of two for every criteria, whilst the control group had a median score 1–1.5 (see “Methods” section and Additional file 1: Table S1).

**Table 2:** Study outcomes for the Patient Pathway Pharmacist Intervention group compared with the control group

Variable	Intervention, *n* = 58	Control, *n* = 50	*P*-value^a^
Discharge summary score, mean (SD)	12.3 (2.3)	7.2 (1.7)	< 0.0001^b^
*Potentially inappropriate medications (PIMs)*^g^			
Mean, at admission (SD)	1.36 (1.6)	1.94 (1.7)	0.131^b^
Mean, at discharge (SD)	1.00 (1.2)^i^	1.83 (1.5)	0.002^b^
Relative change Mean (95% CI)	− 0.36^i^ (− 0.56, − 0.17)	− 0.10 (− 0.40, 0.19)	0.136^b^
*P*-value^c^	< 0.001	0.481	
Between group change in number of PIMs at discharge, mean (95%CI)	− 0.44 (− 0.72, − 0.15)		0.003^d^
*Standard pharmacotherapy*^h^			
Antibiotic prophylaxis during surgery (%)	58/58 (100)	50/50 (100)	
Laxatives with regular opioids (%)	42/58 (72)	17/49^j^ (35)	< 0.001^e^
Osteoporosis treatment at discharge (%)	55/57^i^ (96)	8/50 (16)	< 0.001^e^
Thrombosis prevention at discharge (%)	57/57^i^ (100)	50/50 (100)	
*Readmission rate*			
30 days	11^i^	11	0.703^e^
31–90 days	3	1	0.621^f^
*Mortality*			
Within 30 days	3	2	0.999^f^
31–90 days	7	5	0.999^f^

*IQR*—interquartile range (25th and 75th percentile), *SD*—standard deviation

^a^*P*-value for differences between control group and intervention group given by ^b^Two sample independent student’s *t*-test

^c^Paired sample student’s t-test

^d^ANCOVA (*r*^2^ = 0.75)

^e^Pearson’s Chi Square-test

^f^Fisher’s exact test

^g^Potentially inappropriate medications registered using the Norwegian translation of STOPP criteria, version 2) for patients ≥ 65 years [47, 51]. Mean Relative Change = *n*(discharge) − *n*(admission) per patient. Between group change in number of PIMs at discharge, given by ANCOVA, with PIMs at admission as a covariate

^h^Standard pharmacotherapy found in the medication list at discharge and/or hospital medication chart as appropriate, according to international guidelines for antibiotic prophylaxis during surgery [45], laxatives with regular opioids [47], osteoporosis treatment at discharge [46], and thrombosis prevention[48]

^i^One patient died during hospitalisation and was included in the 30 days mortality, but did not have a discharge summary and was not eligible for readmission or PIMs at discharge

^j^Missing data for one patient

For secondary outcomes, patients in the intervention group had significantly less PIMs at discharge. In both groups, the most frequent STOPP criteria were missing indication, regular use of benzodiazepines, and hypnotic z-medications, and the most frequent PIMs were zopiclone and oxazepam. All patients received antibiotic prophylaxis pre-/interoperatively [45], and thrombosis prevention post-surgery [48]. There was a significantly higher proportion of patients in the intervention group that received prophylactic laxative when opioids were prescribed (72 vs. 35%, *p* < 0.001). This was also the case for patients receiving treatment for osteoporosis at discharge (96 vs. 16%, *p* < 0.001). The higher proportion of patients receiving standard pharmacotherapy was reflected in the higher number of medications at discharge, for which the patients in the intervention group had, on average, 1.42 (95% CI 0.61, 2.23) more medications at discharge. There were no difference between the two groups in readmission rates or death within 30 or 90 days.

### Implementation evaluation

Overall, most patients in the intervention group received the intervention steps per protocol (Table 3). Some deviations from protocol were observed; eight patients were not provided medication information for the discharge summary (step C, Fig. 1) and one patient did not receive medication reconciliation at admission to rehabilitation institution (step D). The mean time for medication reconciliation at admission to rehabilitation institution (step D) was 2.2 days after discharge from hospital. For medication reconciliation (*step E*) and review (step F) post-discharge, the steps were delayed compared to the protocol: from 21–42 days to 40–81 days. For the in-hospital medication review, a total of 110 PIMs were found, recommendations on how to handle the PIMs were given and 57 PIMs (52%) were resolved after the medication review. For the post-discharge medication review, 53 PIMs were detected, and after recommendations on how to handle the PIMs, nine PIMs were resolved (17%).

**Table 3:** Delivery of each intervention step in the Patient Pathway Pharmacist study

Intervention step^a^	Patients receiving step, *n* (%)	Time since discharge, mean/median days (SD or IQR)
*In-hospital*		
Medication reconciliation, admission (*step A*)	58/58 (100)	
Medication review, in-hospital *(step B)*	58/58 (100)	
Medication information in the discharge summary *(step C)*	49/57^b^ (86)	
*Post-discharge*		
Medication reconciliation, at admission to rehabilitation setting *(step D)*	56/57^c^ (98)	2.2 days (1.6) (range 0–7 days)
Medication reconciliation, post-discharge (*step E*)	52/52^d^ (100)	44 days (42–59) (range 40–81)
Medication review, post-discharge* (step F)*	52/52^d^ (100)	44 days (42–59) (range 40–81)

^a^The intervention step lettering is a reference to Fig. 1, which includes details

^b^The medical doctor responsible for discharge used the medication information for the discharge summary in 46/49 cases (94%). The medication information was not written for nine patients due to pharmacist absence (*n* = 2), reason not described (*n* = 3), and the orthopaedist declined to use, or had already written, the medication list (*n* = 4)

^c^One patient did not receive a medication reconciliation at admission to rehabilitation institution for an unknown reason

^d^One patient declined to participate in the post-discharge intervention and four patients had died, with the remaining 52 patients being eligible for post-discharge intervention

## Discussion

The Patient Pathway Pharmacist intervention group showed superior medication management and pharmacotherapy, compared to the control group, through higher quality of medication information in discharge summaries, a lower number of PIMs and more anti-osteoporosis treatment and laxatives. The intervention had, however, no impact on readmission rate or mortality. The intervention was successfully implemented for nearly all included hip fracture patients in both primary and secondary care settings. We interpret the findings to be in favour of hip fracture patients’ safety.

A higher quality of discharge summaries, as seen for the intervention patients, is important to avoid disrupted transfer of medication information and the consequential medication errors. Previous studies have shown that low quality or missing discharge summaries were associated with rehospitalisation [55, 56]. The results from our control group were comparable to observational studies that investigated the quality of discharge summaries [19, 20]. These studies expressed the need for improvement measures, such as electronic medication management systems and involving clinical pharmacists. When a clinical pharmacist provided discharge services, the medication information in discharge summaries improved [29]; a finding supported by this study. Other interventions to improve handover are introducing discharge summary templates and educational training. These strategies have improved discharge summary quality score and timeliness of delivery [15, 57–59]. In comparison with these interventions, we used a strategy that did not increase the demand on hospital doctors. Clinical pharmacists have medications as their expertise and could be a natural successor undertaking these tasks. We believe our results may indicate reduced doctors’ workload in-hospital by performing medication reconciliation and preparing the medication information in the discharge summary, in addition to support robustness of the healthcare system [60, 61].

Intervention patients’ pharmacotherapy improved through medication reviews, by a lower number of PIMs and higher proportion of patients receiving osteoporosis pharmacotherapy at hospital discharge. Reducing the number of PIMs is beneficial to patients as PIMs are associated with new falls and fractures [23], mortality [25], rehospitalisation [62], and adverse drug reactions [63]. Previous studies have described prevalent use of PIMs in hip fracture patients, such as falls-risk increasing drugs [23, 64]. Our results are comparable to previously described clinical pharmacist initiatives, which have reduced PIMs in older inpatients [65], outpatient clinics [66], and hip fracture patients [35]. Through less PIMs and a higher proportion receiving osteoporosis therapy, the intervention patients may have experienced fewer fractures and a lower mortality if observed for a longer period than in this study, e.g. the time seen for bisphosphonate effect; 1–6 years [67, 68]. However, this needs to be verified by future studies.

To the best of our knowledge, we are the first to describe a successful implementation of a clinical pharmacist service designed to follow hip fracture patients throughout the patient pathway, to ensure seamless transfer. This was a key difference from prior clinical pharmacy studies which have focused on a single context, such as ward specific inpatients [69], outpatients [70], transition points [71], or one diagnosis tied to one setting [72]. Although, there are examples of studies following the patient through hospital care with follow-up in primary care for a wider group of patients than hip fracture only [31, 73]. An alternative profession that could have contributed with a similar intervention, are geriatricians which could have been a Patient Pathway Geriatrician. The post-discharge steps of the Patient Pathway Pharmacist intervention was delayed to 6–12 weeks after discharge. The reason for delay was undocumented, but our experience was that the delay was a consequence of rehabilitation and increased care need for hip fracture patients, which typically lasts 12 weeks if prolonged [74].

The number of PIMs could be reduced through medication reviews at two intervention points; in-hospital and in the patients’ home after discharge. The implementation evaluation showed PIMs to be reduced by 50% through the hospital medication review, but only by 17% in the home setting. We hypothesise that this result has two explanations. First, the PIMs at hospital were the most grave and obvious to discontinue. Thus, in home setting, the PIMs available to discontinue may have been the PIMs that were difficult to stop without an alternative pharmacotherapy or elaborate follow-up from a healthcare professional. Second, the communication between pharmacist and primary care doctor might have been less successful over the telephone (e.g. understanding the pharmacist role or knowledge, or time shortage). Although barriers for de-prescribing is complex and multifactorial [75], the second reason is comparable to previously described barriers for successful implementation of clinical pharmacist services (“interpersonal skills and relationships” and “working patterns”) [76].

A strength of our study was the contribution towards bridging the gap of silo-organised healthcare systems, which often act as communication barriers between different care settings, thus mediating seamless care transition and continuity. Furthermore, the discharge summary score contained the most clinically relevant criteria for safe medication management in the next care setting, as it was in accordance with international guidelines [14, 18, 77, 78], previous literature [19, 20, 79], and the Norwegian quality score [49]. Additionally, the discharge summary results showed high reliability. The pre-intervention controls were comparable to the intervention groups in regard to being from the same study site, personal characteristics, similar inclusion and exclusion criteria, investigating the same outcome measures, and recruited immediately before the inclusion to the intervention group.

We chose not to conduct the study as a randomised controlled trial (RCT) due to the high risk of contamination bias as the study hospital has one orthopaedic ward only, and most often the same doctor is responsible for the ward round in all hip fracture patients [80, 81]. Including multiple centres in a cluster RCT was not possible due to resource limitations. We acknowledge that the single centre, nonrandomised design is a limitation to our study as it challenges the generalisability, and introduce a causality discussion and possibility of bias. Although RCT’s are considered gold standard in terms of causality, the majority of Bradford-Hill criteria support causality in our study (strength, temporality, plausibility, coherence, and analogy) [82]. Particularly important, the main difference between the intervention and control group was the intervention itself; we were not aware of external changes (e.g. in organisation, staffing or routines) during the study period. The three months prior to intervention was chosen for the control group to reduce time-dependent bias, as a longer temporal distance may introduce external changes. Albeit, the intervention group patients were admitted during autumn and winter, seasons associated with increased incidence and mortality risk [83–85]. Any selection bias was counteracted by consistently including the last patient admitted, but as only patients in the intervention group were able to decline participation some skewness may have occurred. For instance, a greater proportion of patients in the intervention group, although non-significantly, lived in private homes before admission and had lower CCI, indicating slightly healthier intervention group. In contrast, patients who died during hospitalisation were excluded from the pre-intervention control group due to the lack of discharge summaries, which may have resulted in a slightly healthier control group. The potential selection bias would only affect readmission rate and mortality. Lastly, we consider the involvement of only one intervention pharmacist (BTH) to be a limitation of our study. However, working by the structured IMM method support generalisation of findings to any pharmacist following this method.

By showing implementation of the interventions steps and improvement in discharge summary score, we claim the Patient Pathway Pharmacist intervention is feasible to be delivered in a clinical setting, and contributed to safe transfer of medication information and appropriate pharmacotherapy in all care settings, thus supporting patient safety [15, 30]. We regard the result to be clinically relevant as the discharge summary score tool address criteria paramount for safe medication management. Future studies are needed to explore the generalisability of the Patient Pathway Pharmacist intervention, investigate the impact it may have on readmission rate and mortality, preferably as primary outcome measures. Additionally, with an increased number of participants, it would be possible to study impact in different subgroups, such as patients living at home and patients living in nursing homes prior to fracture. As a higher proportion of patients in the intervention group received osteoporosis treatment, which has effects that may last 5–10 years, the follow-up period should ideally be several years [86, 87]. In addition, the design should enable the post-discharge intervention steps to be compared with the control group, such as number of PIMs and, potentially, a patient reported outcome measure. The ideal design would presumably be multi-centre studies, using cluster-randomised RCT or a stepped-wedge design.

## Conclusion

The innovative Patient Pathway Pharmacist intervention was successfully implemented for nearly all patients in primary and secondary care settings. The intervention contributed to hip fracture patient safety through higher quality medication information in the discharge summary, lower number of PIMs, and higher proportion receiving standard pharmacotherapy. The intervention had no effect on the secondary outcomes readmission rate or mortality. Future studies are needed to explore the generalisability of this intervention.

## Supplementary Information

**Additional file 1**: Scoring tool for the quality of the medication information in the discharge summary and reliability test.

**Additional file 2**: The included STOPP-2 (Screening tool for older person’s prescriptions, version 2) criteria.

## Data Availability

The datasets generated and analysed during the current study are not publicly available due to restrictions in the approval given by the Norwegian Centre for Research Data.

## Abbreviations

ANCOVA: Analysis of covariance
CCI: Charlson’s Comorbidity Index
CI: Confidence interval
ICC: Intraclass correlation coefficient
IQR: Interquartile range
PIMs: Potentially inappropriate medications
RCT: Randomised controlled trial
STOPP: Screening tool of older people’s prescriptions
SD: Standard deviation
