# Supplementary material for: Clinical pharmacist intervention to improve medication safety for hip fracture patients through secondary and primary care settings: a nonrandomised controlled trial
Source: J Orthop Surg Res. 2023 Jun 13;18:434. doi: 10.1186/s13018-023-03906-2 (PMC10265814; doi:10.1186/s13018-023-03906-2)
Supplement: Supplementary file 1 — Additional file 1: Scoring tool for the quality of the medication information in the discharge summary and reliability test. [file 13018_2023_3906_MOESM1_ESM.pdf]

ADDITIONAL FILE 1: SCORING TOOL FOR THE QUALITY OF THE MEDICATION INFORMATION IN THE DISCHARGE SUMMARY AND RELIABILITY TEST

Supplemental Material to:

Henriksen, BT[*1,2,3], Krogseth M[4], Andersen RD[5,6], Davies MN[1], Nguyen CT[1,7], Mathiesen L[3], Andersson Y[1]. **Clinical pharmacist intervention to improve medication safety for hip fracture patients through secondary and primary care settings: A nonrandomised controlled trial.** Journal of Orthopaedic Surgery and Research. 2023.

[1]Research department, Hospital Pharmacies Enterprise, South Eastern Norway, Tonsberg, Norway

[2]Division of Surgery, Vestfold Hospital Trust, Tonsberg, Norway

[3]Department of Pharmacy, Faculty of Mathematics and Natural Sciences, University of Oslo, Oslo, Norway

[4]Old Age Psychiatry Research Network, Telemark Vestfold, Vestfold Hospital Trust, Tonsberg, Norway

[5]Department of Research, Telemark Hospital Trust, Skien, Norway

[6]Institute of Health and Society, Research Centre for Habilitation and Rehabilitation Models & Services (CHARM), Faculty of Medicine, University of Oslo, Oslo, Norway

[7]Department of Pharmacy, Faculty of Health Sciences, UiT The Arctic University of Tromso, Tromso, Norway

[*]Corresponding author. *Email:* Ben.Tore.Henriksen@Sykehusapotekene.no. *ORCiD:* 0000-0001-7250-7597

The tool used in this study to measure the quality of medication information in discharge summaries, including median values for each criteria and mean total score, is shown in table S1. All discharge summaries were independently rated by experienced clinical pharmacists and an MSc Pharmacy student. For the intervention patients, the discharge summaries were rated by the intervention pharmacist, *initials*, during the intervention period, and by an external rater (experienced clinical pharmacist). The control group's discharge summaries were rated by (*initials*) during data collection and afterwards by *initials*. The inter-rater agreement and reliability of the discharge summary score were calculated using weighted kappa for intra-criteria reliability and intraclass correlation coefficient (ICC) (two-way mixed effect model, absolute agreement, average measure) [1, 2] for the total score (table S2). For the intervention group, the reliability for total score was excellent (ICC 0.96 (95%CI 0.93 , 0.98), $p<0.001$), and the agreement for each criteria was 88.6-98.3%, with weighted kappa ranging from moderate to almost perfect (κ_w 0.56-0.89). For the control group, the reliability for total score was good [2], and the agreement for each criteria was 69.0-96.0%, with weighted kappa ranging from slight to almost perfect (κ_w 0.20 – 0.91) [3]. The highest agreement was for criteria frequencies and indication for the intervention and control group, respectively. The highest kappa-value was for criteria generic names and indications for the intervention and control group, respectively. The lowest agreement and kappa-value were for criteria 'reasons for medication changes' in both groups. The reliability test results were controlled by an external statistician who were blinded for group and raters.

Table S1: Median score for each criteria and total of the discharge summary scoring tool for the quality of the medication information in the discharge summary for the Patient Pathway Pharmacist Intervention group compared with the pre-intervention control group

		**Intervention,** n=57		**Control,** n=50		**P-value**[a]
**#**	**Criteria** *Points*[b]*: yes=2, partially=1, no=0*	**Median (IQR)**	**Range**	**Median (IQR)**	**Range**	
1	Are the reasons for medication changes at discharge included in the discharge summary?	2 (1.5-2)	0-2	1 (0.5-1.5)	0-2	<0.0001
2	Are generic names included in the discharge summary?	2 (1-2)	0-2	1 (1-1)	0-1	<0.0001
3	Are medication formulations included?	2 (2-2)	0-2	1 (0.5-1)	0-2	<0.0001
4	Are medication doses included?	2 (2-2)	1-2	1.25 (1-2)	0.5-2	<0.0001
5	Are frequencies included?	2 (2-2)	1-2	1.5 (1-2)	1-2	<0.0001
6	Are indications for use included?	2 (1-2)	0-2	1 (0-1)	0-1	<0.0001
7	Are categories for changed medication described? [c]	2 (2-2)	0-2	1 (1-1.5)	0-2	<0.0001
		**Mean (SD)**		**Mean (SD)**		
**Total discharge summary score**	*Maximum possible score: 14*	12.32 (2.31)	5-14	7.23 (1.69)	3.5-10.5	<0.0001

Abbreviations: SD; Standard Deviation, IQR; Interquartile range (presented as the 25th – 75th percentile).
[a]P-values given by two-sample independent student's t-test for total discharge summary score and Mann-Whitney U-test for each criteria by comparing control and intervention group.
[b]Discharge summary score based on the Norwegian Safety Programme's scoring tool for the quality of the medication information in the discharge summary [4]. The score for each criteria was based on whether the information was present for all medications (two points), for at least one medication (one point), or not at all (zero points), hence the final score ranged from 0-14. The discharge summary score was calculated as the mean value between the two raters for control and intervention group.
[c]Refers to the category for changed medication (new, stopped, changed, short course), where one of the options is to be stated in front or behind each relevant medication in the medication list.

Table S2: Summary and intraclass correlation coefficient of total discharge summary score for the intervention group and pre-intervention control group

	Intervention (n = 57)			Control (n = 50)		
	Rater A	Rater B	*p-value*[a]	Rater C	Rater B	*p-value*[a]
Mean (SD)	12.19 (2.24)	12.44 (2.46)	*0.0513*	7.72 (1.57)	6.74 (2.06)	*<0.0001*
[95% CI]	[11.60-12.79]	[11.78-13.09]		[7.28-8.16]	[6.16-7.33]	
Range	5-14	5-14		4-11	2-11	
ICC	0.959		*0.001*	0.835		*<0.0001*
[95% CI]	[0.91-0.98]			[0.71-0.91]		

Abbreviations: CI; Confidence Interval, ICC; Intraclass Correlation Coefficient, SD; Standard Deviation.
[a]Testing for differences in means using paired-sample student's t-test.
Rater A: External rater (experienced clinical pharmacist), discharge summaries scored in 2022.
Rater B: The Patient Pathway Pharmacist, discharge summaries scored in 2018-2019 (intervention group) and 2022 (control group).
Rater C: Master student in pharmacy, discharge summaries scored occurred in 2018-2019.
Intraclass Correlation Coefficient: Two-way mixed effect model, absolute agreement, average measure [1, 2].

REFERENCES

1. McGraw KO, Wong SP. Forming Inferences About Some Intraclass Correlation Coefficients. Psychol Methods 1996;1(1):30-46.
2. Koo TK, Li MY. A Guideline of Selecting and Reporting Intraclass Correlation Coefficients for Reliability Research. J Chiropr Med 2016;15(2):155-63.
3. Landis JR, Koch GG. The Measurement of Observer Agreement for Categorical Data. Biometrics 1977;33(1):159-74.
4. Nasjonalt pasientsikkerhetsprogram I trygge hender 24-7. Tiltakspakke for samstemming av legemiddellister. [Internet]. Oslo: Helsedirektoratet [Norwegian Directorate of Health]; 2015 [updated [date unknown]; cited 2022 Dec 13]. Available from: https://www.itryggehender24-7.no/reduser-pasientskader/legemiddelrelaterte-skader/legemiddelsamstemming-og-legemiddelgjennomgang/_/attachment/download/33b5eb0c-4f62-4d42-862f-b9f95f09423c:12bcc5de3b8e1ff2e4bdb9937951300d8d6f35cc/tiltakspakke-for-samstemming-av-legemiddellister.pdf. Norwegian.
