# Supplementary material for: Clinical pharmacist intervention to improve medication safety for hip fracture patients through secondary and primary care settings: a nonrandomised controlled trial
Source: J Orthop Surg Res. 2023 Jun 13;18:434. doi: 10.1186/s13018-023-03906-2 (PMC10265814; doi:10.1186/s13018-023-03906-2)
Supplement: Supplementary file 2 — Additional file 2: The included STOPP-2 (Screening tool for older person’s prescriptions, version 2) criteria. [file 13018_2023_3906_MOESM2_ESM.pdf]

ADDITIONAL FILE 2: STOPP-2 (SCREENING TOOL FOR OLDER PERSON'S PRESCRIPTIONS, VERSION 2)

Supplemental Material to:

Henriksen, BT[*1,2,3], Krogseth M[4], Andersen RD[5,6], Davies MN[1], Nguyen CT[1,7], Mathiesen L[3], Andersson Y[1]. **Clinical pharmacist intervention to improve medication safety for hip fracture patients through secondary and primary care settings: A nonrandomised controlled trial.** Journal of Orthopaedic Surgery and Research. 2023.

[1]Research department, Hospital Pharmacies Enterprise, South Eastern Norway, Tonsberg, Norway

[2]Division of Surgery, Vestfold Hospital Trust, Tonsberg, Norway

[3]Department of Pharmacy, Faculty of Mathematics and Natural Sciences, University of Oslo, Oslo, Norway

[4]Old Age Psychiatry Research Network, Telemark Vestfold, Vestfold Hospital Trust, Tonsberg, Norway

[5]Department of Research, Telemark Hospital Trust, Skien, Norway

[6]Institute of Health and Society, Research Centre for Habilitation and Rehabilitation Models & Services (CHARM), Faculty of Medicine, University of Oslo, Oslo, Norway

[7]Department of Pharmacy, Faculty of Health Sciences, UiT The Arctic University of Tromso, Tromso, Norway

[*]Corresponding author. *Email:* Ben.Tore.Henriksen@Sykehusapotekene.no. *ORCiD:* 0000-0001-7250-7597

Only STOPP criteria that could be assessed by clinical data available in patient records were included, causing 24 of the total 81 categories to be excluded (table S3). Examples of reasons for excluding criteria were if the criteria depended on the length of treatment, such as criteria K2 (benzodiazepines used for more than 4 weeks), and if the criteria depended on whether the medication was the first choice treatment, such as criteria D2 (tricyclic antidepressants as the first choice for depression).

Table S3: List of STOPP-2 (Screening Tool for Older Person's Prescriptions, version 2) criteria included in the Patient Pathway Pharmacist study

The following prescriptions are potentially inappropriate to use in patients aged 65 years and older.

Section A: Indication of medication

1. Any drug prescribed without an evidence-based clinical indication.
3. Any duplicate drug class prescription e.g. two concurrent NSAIDs, SSRIs, loop diuretics, ACE inhibitors, anticoagulants (optimisation of monotherapy within a single drug class should be observed prior to considering a new agent).

Section B: Cardiovascular System

3. Beta-blocker in combination with verapamil or diltiazem (risk of heart block).
4. Beta blocker with bradycardia (< 50/min), type II heart block or complete heart block (risk of complete heart block, asystole).
8. Thiazide diuretic with current significant hypokalaemia (i.e. serum K+ < 3.0 mmol/l), hyponatraemia (i.e. serum Na+ < 130 mmol/l) hypercalcaemia (i.e. corrected serum calcium > 2.65 mmol/l) or with a history of gout (hypokalaemia, hyponatraemia, hypercalcaemia and gout can be precipitated by thiazide diuretic)
9. Loop diuretic for treatment of hypertension with concurrent urinary incontinence (may exacerbate incontinence).
11. ACE inhibitors or Angiotensin Receptor Blockers in patients with hyperkalaemia.

Section C: Antiplatelet/Anticoagulant Drugs

1. Long-term aspirin at doses greater than 160mg per day (increased risk of bleeding, no evidence for increased efficacy).
2. Aspirin with a past history of peptic ulcer disease without concomitant PPI (risk of recurrent peptic ulcer).
4. Aspirin plus clopidogrel as secondary stroke prevention, unless the patient has a coronary stent(s) inserted in the previous 12 months or concurrent acute coronary syndrome or has a high grade symptomatic carotid arterial stenosis (no evidence of added benefit over clopidogrel monotherapy)
5. Aspirin in combination with vitamin K antagonist, direct thrombin inhibitor or factor Xa inhibitors in patients with chronic atrial fibrillation (no added benefit from aspirin)
6. Antiplatelet agents with vitamin K antagonist, direct thrombin inhibitor or factor Xa inhibitors in patients with stable coronary, cerebrovascular or peripheral arterial disease (No added benefit from dual therapy).
7. Ticlopidine in any circumstances (clopidogrel and prasugrel have similar efficacy, stronger evidence and fewer side-effects).
10. NSAID and vitamin K antagonist, direct thrombin inhibitor or factor Xa inhibitors in combination (risk of major gastrointestinal bleeding).
11. NSAID with concurrent antiplatelet agent(s) without PPI prophylaxis (increased risk of peptic ulcer disease)

Table S3 cont.

Section D: Central Nervous System and Psychotropic Drugs

1. TriCyclic Antidepressants (TCAs) with dementia, narrow angle glaucoma, cardiac conduction abnormalities, prostatism, or prior history of urinary retention (risk of worsening these conditions).
3. Neuroleptics with moderate-marked antimuscarinic/anticholinergic effects (chlorpromazine, clozapine, flupenthixol, fluphenzine, pipothiazine, promazine, zuclopenthixol) with a history of prostatism or previous urinary retention (high risk of urinary retention).
4. Selective serotonin re-uptake inhibitors (SSRI's) with current or recent significant hyponatraemia i.e. serum Na+ < 130 mmol/l (risk of exacerbating or precipitating hyponatraemia).
6. Antipsychotics (i.e. other than quetiapine or clozapine) in those with parkinsonism or Lewy Body Disease (risk of severe extra-pyramidal symptoms)
7. Anticholinergics/antimuscarinics to treat extra-pyramidal side-effects of neuroleptic medications (risk of anticholinergic toxicity),
8. Anticholinergics/antimuscarinics in patients with delirium or dementia (risk of exacerbation of cognitive impairment).
10. Neuroleptics as hypnotics, unless sleep disorder is due to psychosis or dementia (risk of confusion, hypotension, extra-pyramidal side effects, falls).
11. Acetylcholinesterase inhibitors with a known history of persistent bradycardia (< 60 beats/min.), heart block or recurrent unexplained syncope or concurrent treatment with drugs that reduce heart rate such as beta-blockers, digoxin, diltiazem, verapamil (risk of cardiac conduction failure, syncope and injury).
13. Levodopa or dopamine agonists for benign essential tremor (no evidence of efficacy)
14. First-generation antihistamines (safer, less toxic antihistamines now widely available).

Section E: Renal System. The following drugs are potentially inappropriate in older people with acute or chronic kidney disease with renal function below particular levels of eGFR (refer to summary of product characteristics datasheets and local formulary guidelines)

1. Digoxin at a long-term dose greater than 125µg/day if eGFR < 30 ml/min/1.73m2 (risk of digoxin toxicity if plasma levels not measured).
2. Direct thrombin inhibitors (e.g. dabigatran) if eGFR < 30 ml/min/1.73m2 (risk of bleeding)
3. Factor Xa inhibitors (e.g. rivaroxaban, apixaban) if eGFR < 15 ml/min/1.73m2 (risk of bleeding)
4. NSAID's if eGFR < 50 ml/min/1.73m2 (risk of deterioration in renal function).
5. Colchicine if eGFR < 10 ml/min/1.73m2 (risk of colchicine toxicity)
6. Metformin if eGFR < 30 ml/min/1.73m2 (risk of lactic acidosis).

Section F: Gastrointestinal System

1. Prochlorperazine or metoclopramide with Parkinsonism (risk of exacerbating Parkinsonian symptoms).
4. Oral elemental iron doses greater than 200 mg daily (e.g. ferrous fumarate> 600 mg/day, ferrous sulphate > 600 mg/day, ferrous gluconate> 1800 mg/day; no evidence of enhanced iron absorption above these doses).

Section G: Respiratory System

1. Theophylline as monotherapy for COPD (safer, more effective alternative; risk of adverse effects due to narrow therapeutic index).
2. Systemic corticosteroids instead of inhaled corticosteroids for maintenance therapy in moderate-severe COPD (unnecessary exposure to long-term side-effects of systemic corticosteroids and effective inhaled therapies are available).
3. Anti-muscarinic bronchodilators (e.g. ipratropium, tiotropium) with a history of narrow angle glaucoma (may exacerbate glaucoma) or bladder outflow obstruction (may cause urinary retention).
4. Non-selective beta-blocker (whether oral or topical for glaucoma) with a history of asthma requiring treatment (risk of increased bronchospasm).

Table S3 cont.

Section H: Musculoskeletal System

1. Non-steroidal anti-inflammatory drug (NSAID) other than COX-2 selective agents with history of peptic ulcer disease or gastrointestinal bleeding, unless with concurrent PPI or H2 antagonist (risk of peptic ulcer relapse).
2. NSAID with severe hypertension (risk of exacerbation of hypertension) or severe heart failure (risk of exacerbation of heart failure).
5. Corticosteroids (other than periodic intra-articular injections for mono-articular pain) for osteoarthritis (risk of systemic corticosteroid side-effects).
7. COX-2 selective NSAIDs with concurrent cardiovascular disease (increased risk of myocardial infarction and stroke)
8. NSAID with concurrent corticosteroids without PPI prophylaxis (increased risk of peptic ulcer disease)
9. Oral bisphosphonates in patients with a current or recent history of upper gastrointestinal disease i.e. dysphagia, oesophagitis, gastritis, duodenitis, or peptic ulcer disease, or upper gastrointestinal bleeding (risk of relapse/exacerbation of oesophagitis, oesophageal ulcer, oesophageal stricture)

Section I: Urogenital System

1. Antimuscarinic drugs with dementia, or chronic cognitive impairment (risk of increased confusion, agitation) or narrow-angle glaucoma (risk of acute exacerbation of glaucoma), or chronic prostatism (risk of urinary retention).
2. Selective alpha-1 selective alpha blockers in those with symptomatic orthostatic hypotension or micturition syncope (risk of precipitating recurrent syncope)

Section J. Endocrine System

1. Sulphonylureas with a long duration of action (e.g. glibenclamide, chlorpropamide, glimepiride) with type 2 diabetes mellitus (risk of prolonged hypoglycaemia).
2. Thiazolidenediones (e.g. rosiglitazone, pioglitazone) in patients with heart failure (risk of exacerbation of heart failure)
4. Oestrogens with a history of breast cancer or venous thromboembolism (increased risk of recurrence).
5. Oral oestrogens without progestogen in patients with intact uterus (risk of endometrial cancer).
6. Androgens (male sex hormones) in the absence of primary or secondary hypogonadism (risk of androgen toxicity; no proven benefit outside of the hypogonadism indication).

Section K: Drugs that predictably increase the risk of falls in older people

1. Benzodiazepines (sedative, may cause reduced sensorium, impair balance).
2. Neuroleptic drugs (may cause gait dyspraxia, Parkinsonism).
3. Vasodilator drugs (e.g. alpha-1 receptor blockers, calcium channel blockers, long-acting nitrates, ACE inhibitors, angiotensin I receptor blockers,) with persistent postural hypotension i.e. recurrent drop in systolic blood pressure ≥ 20mmHg (risk of syncope, falls).
4. Hypnotic Z-drugs e.g. zopiclone, zolpidem, zaleplon (may cause protracted daytime sedation, ataxia).

Section L: Analgesic Drugs

2. Use of regular (as distinct from PRN) opioids without concomitant laxative (risk of severe constipation).
3. Long-acting opioids without short-acting opioids for break-through pain (risk of persistence of severe pain)

Section N: Antimuscarinic/Anticholinergic Drug Burden

1. Concomitant use of two or more drugs with antimuscarinic/anticholinergic properties (e.g. bladder antispasmodics, intestinal antispasmodics, tricyclic antidepressants, first generation antihistamines) (risk of increased antimuscarinic/anticholinergic toxicity)

Criteria A2, B1, B2, B5, B6, B7, B10, B12, B13, C3, C8, C9, D2, D5, D9, D12, F2, F3, G5, H3, H4, H6, J3, and L1 in the original STOPP-2 was excluded. *Reference: O'Mahony D, O'Sullivan D, Byrne S, O'Connor MN, Ryan C, Gallagher P. STOPP/START criteria for potentially inappropriate prescribing in older people: version 2. Age and Ageing. 2015;44(2):213-8.*
